# *Let-7 *microRNAs are developmentally regulated in circulating human erythroid cells

**DOI:** 10.1186/1479-5876-7-98

**Published:** 2009-11-25

**Authors:** Seung-Jae Noh, Samuel H Miller, Y Terry Lee, Sung-Ho Goh, Francesco M Marincola, David F Stroncek, Christopher Reed, Ena Wang, Jeffery L Miller

**Affiliations:** 1 Molecular Medicine Branch, National Institute of Diabetes, Digestive, and Kidney Diseases, National Institutes of Health, Bethesda, Maryland, USA; 2 Department of Transfusion Medicine, Clinical Center, National Institutes of Health, Bethesda, Maryland, USA; 3 National Naval Medical Center, Department of Obstetrics and Gynecology, Bethesda, Maryland, USA; 4 National Cancer Center, Goyang-si, Gyeonggi-do, Republic of Korea

## Abstract

**Background:**

MicroRNAs are ~22nt-long small non-coding RNAs that negatively regulate protein expression through mRNA degradation or translational repression in eukaryotic cells. Based upon their importance in regulating development and terminal differentiation in model systems, erythrocyte microRNA profiles were examined at birth and in adults to determine if changes in their abundance coincide with the developmental phenomenon of hemoglobin switching.

**Methods:**

Expression profiling of microRNA was performed using total RNA from four adult peripheral blood samples compared to four cord blood samples after depletion of plasma, platelets, and nucleated cells. Labeled RNAs were hybridized to custom spotted arrays containing 474 human microRNA species (miRBase release 9.1). Total RNA from Epstein-Barr virus (EBV)-transformed lymphoblastoid cell lines provided a hybridization reference for all samples to generate microRNA abundance profile for each sample.

**Results:**

Among 206 detected miRNAs, 79% of the microRNAs were present at equivalent levels in both cord and adult cells. By comparison, 37 microRNAs were up-regulated and 4 microRNAs were down-regulated in adult erythroid cells (fold change > 2; p < 0.01). Among the up-regulated subset, the *let*-7 miRNA family consistently demonstrated increased abundance in the adult samples by array-based analyses that were confirmed by quantitative PCR (4.5 to 18.4 fold increases in 6 of 8 *let-7 *miRNA). Profiling studies of messenger RNA (mRNA) in these cells additionally demonstrated down-regulation of ten let-7 target genes in the adult cells.

**Conclusion:**

These data suggest that a consistent pattern of up-regulation among *let-7 *miRNA in circulating erythroid cells occurs in association with hemoglobin switching during the fetal-to-adult developmental transition in humans.

## Background

MicroRNA (miRNA) is approximately 22 nucleotide long single-stranded RNA which regulates gene expression through either post-transcriptional gene silencing by pairing to target mRNA to trigger mRNA cleavage, trafficking of mRNA for degradation, or translational repression [1]. MicroRNAs are predicted to target over one-third of the human genome [2]. Regulated expression of miRNA was linked to many physiological processes including developmental timing and neuronal patterning [3]. Gene products that control a broad range of functions including proliferation, differentiation and apoptosis are targeted by miRNA [4,5]. For example, expression of miR-145 is thought to act as a tumor suppressor in normal cells, and miR-145 is under-expressed in breast cancer. Alternatively, over-expression of a separate miRNA named miR-155 is thought to be involved in oncogenesis [6]. Expression of some miRNA is evolutionarily-conserved including the *let-7 *miRNA family. Experimental findings suggest that *let-7 *miRNAs play major roles in growth and development [7]. Based upon involvement of *let-7 *miRNA in the larval-to-adult transition in *C. elegans *and the juvenile-to-adult transition in *Drosophila*, a similar function for *let-7 *miRNA in mammalian development is being explored [8].

Birth defines the developmental transition from fetal to extra-uterine life in humans. Post-natal life necessitates the development or function of several organ systems that maintain those functions into adulthood. The loss of placental function necessitates pulmonary function and atmospheric respiration for adequate tissue oxygenation and survival of the host. Tissue oxygenation is accomplished during this developmental period via hemoglobin in erythrocytes that complete the placental or pulmonary circuits [9]. Human hemoglobin is a heterotetrameric metalloprotein composed with four globin chains; two of alpha chains (α1, α2, ζ, μ, and θ) and two of beta chains (β, δ, G-γ, A-γ, and ε). Each globin molecule binds one heme molecule [10]. In humans and other large mammals, the perinatal period defines a major developmental transition from fetal-to-adult hemoglobin types in erythroid cells [11]. Hemoglobin composition switches around the time of birth from fetal hemoglobin (HbF, α_2_γ_2_) to adult hemoglobin (HbA, α_2_β_2_). Based upon the importance of hemoglobin switching for the clinical development of sickle cell anemia and thalassemias, this developmental hemoglobin switching process has been studied extensively. While studies of hemoglobin switching led to fundamental insights regarding gene and protein structure and regulation over the last 50 years, the molecular mechanism(s) for this developmental phenomenon remain elusive. Hemoglobin switching is accomplished via developmentally timed and coordinated changes in globin gene expression. As such, efforts remain focused upon understanding transcription regulation in erythroid cells. Since miRNA represent a new class of transcription regulators in eukaryotic cells, human circulating erythroid cells were used to determine whether fetal-to-adult hemoglobin switching is associated with changes in miRNA abundance patterns.

## Methods

### Preparation of reticulocyte RNA

Studies involving human subjects were approved by the institutional review boards of the National Institute of Diabetes, Digestive, and Kidney Diseases or the National Naval Medical Center. After written informed consent was obtained, peripheral blood or umbilical cord blood was collected from four adult healthy volunteers and four pregnant females. Reticulocyte-enriched pool was obtained by removing plasma, platelets, and white blood cells by centrifugation and filtering as described previously [12]. Total RNA was isolated from the reticulocyte-enriched pool using TRIzol reagent.

### Transcriptome profiling of reticulocytes from cord and adult bloods

Profiles of mRNA expression were analyzed based on total RNA from six cord blood and six adult blood samples using GeneChip^® ^Human Genome U133 Plus 2.0 arrays (Affymetrix) with the same method as previously described [12].

### MicroRNA array analysis

Custom spotted miRNA array V4P4 containing duplicated 713 human, mammalian and viral mature antisense microRNA species (miRBase: http://www.mirbase.org/, version 9.1) plus 2 internal controls with 7 serial dilutions was printed in house (Immunogenetics Laboratory, Department of Transfusion Medicine, Clinical Center, National Institutes of Health). Validation of this platform according to sample input, dye reversal, and labeling method efficiency were optimized for analyses of microRNA species in hematopoietic cells as reported previously [13]. The oligo probes were 5' amine modified and immobilized in duplicate on CodeLink activated slides (GE Healthcare, Piscataway, NJ) via covalent binding. Fluorescent labeled miRNA from total RNA samples was synthesized using miRCURY LNA microRNA Power labeling kit (Exiqon, Woburn, MA) according to manufacturer's protocol. Purified total RNA from four cord blood and four adult RBC was labeled with fluorescent Hy5-dye. Reference total RNA isolated from Epstein-Barr virus (EBV)-transformed lymphoblastoid cell lines were labeled with fluorescent Hy3-dye for comparison. Labeled RNA from sample and reference were co-hybridized to miRNA array at room temperature overnight. After washing, raw intensity data were obtained by scanning the chips with GenePix scanner Pro 4.0 and were normalized by median over entire array. Differentially expressed miRNAs were defined by two-tailed unpaired t-test comparing cord blood group with adult blood group as miRNAs with p-value less than 0.01 and fold change greater than two. All microarray data compiled for this study is MIAME compliant and the raw data has been deposited in a MIAME compliant database (GEO#: GSE17639, GSE17405).

### Quantitative real-time PCR

To confirm the microarray results, quantitative real-time PCR (qPCR) was performed on let-7a through let-7i miRNA members in adult blood vs. cord blood. Complementary DNA specific to each miRNA was generated from total RNA using TaqMan MicroRNA Reverse Transcription Kit (Applied Biosystems) according to manufacturer's protocol and subjected to the real-time PCR reaction using Taqman microRNA assay (Applied Biosystems). Each reaction was performed in triplicate. miR-103 was chosen as the endogenous control for signal normalization across different samples based on the recommendation of previous report [14]. Normalized relative expression level of each miRNA was approximated by calculating 2-ΔCt (ΔCt = Ct_miRNA - Ct_miR-103, Ct: cycle threshold). Variation of mean Ct of miR-103 across four cord blood and four adult blood samples remained low (Avg_Ct = 19.75, Stdev = 1.09).

## Results and Discussion

**Figure 1:**
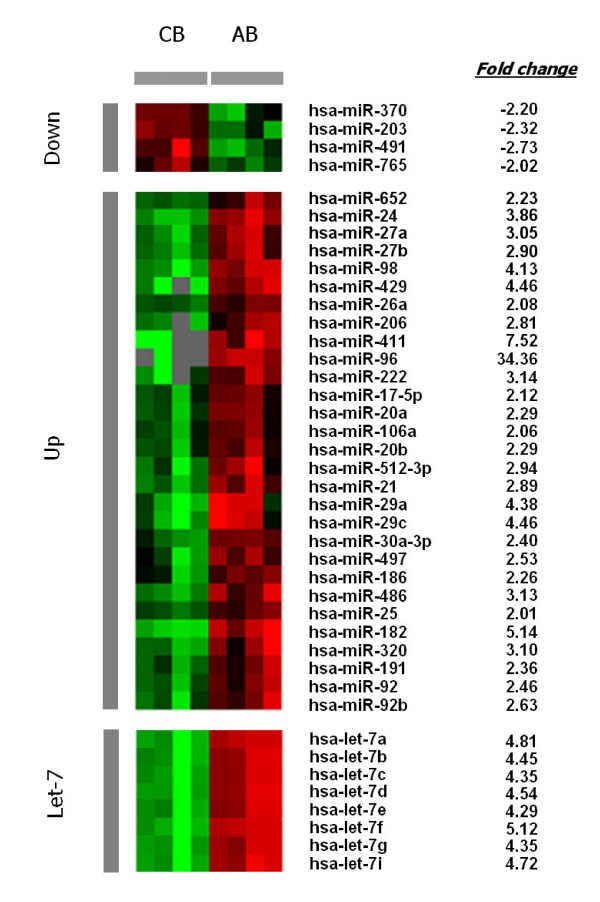
**MicroRNA expression profiles of reticulocytes from cord blood and adult blood samples**. Total RNA was isolated from enucleated reticulocyte-enriched pools from four umbilical cord blood samples (CB) and four adult peripheral blood samples (AB). Raw intensities from each sample were normalized compared to median value over entire array. As shown, miRNA defined as being differentially expressed (*p *< 0.01 and fold change > 2) were grouped into down-regulated (Down), up-regulated (Up), and *let-7 *(Let-7) gene products. Relative abundance patterns are noted as increased (red), decreased (green), unchanged (black), and below the detection limit (grey).

Erythroid cells (reticulocytes and mature erythrocytes) were isolated and purified from blood. The strategies used to isolate the erythroid cells in high purity (>99% erythroid cells in the absence of leukocytes and platelets) were previously described [12]. Total RNA was isolated within 48 hours of collection from fetal (umbilical cord, n = 4) and adult (n = 4) blood sources. Among the 474 human miRNAs spotted on the arrays, 206 were detected in the samples. As defined by p < 0.01 and mean fold change > 2, 41 miRNA species were identified as being differentially expressed in the fetal and adult cells. According to these criteria, only 4 of 41 miRNAs demonstrated significantly down-regulated abundance in the adult cells, and none were down-regulated to levels below a negative three-fold change. The remaining 37 of the 206 human miRNAs were upregulated in abundance in the adult samples. Among the up-regulated subgroup, hsa-miR-96 demonstrated a distinct pattern with a 34.4 fold increase in abundance. Also noteworthy were hsa-miR-411 with a 7.5 fold increase, hsa-miR-182 with a 5.1 fold increase, and hsa-*let-7 *miRNAs with 4.3 to 5.1 fold increases (Figure 1). The unbalanced pattern of up-regulation compared to down-regulation in the adult samples was opposite the pattern of mRNA previously reported among similar erythroid populations [12]. In that study, the fetal erythroid cells were identified as having increased abundance in 103 of 107 differentially regulated mRNAs. The cause of increased abundance of miRNA versus decreased mRNA abundance in the adult cells is unknown, but the pattern is consistent with the general role of miRNA for mRNA degradation.

**Figure 2:**
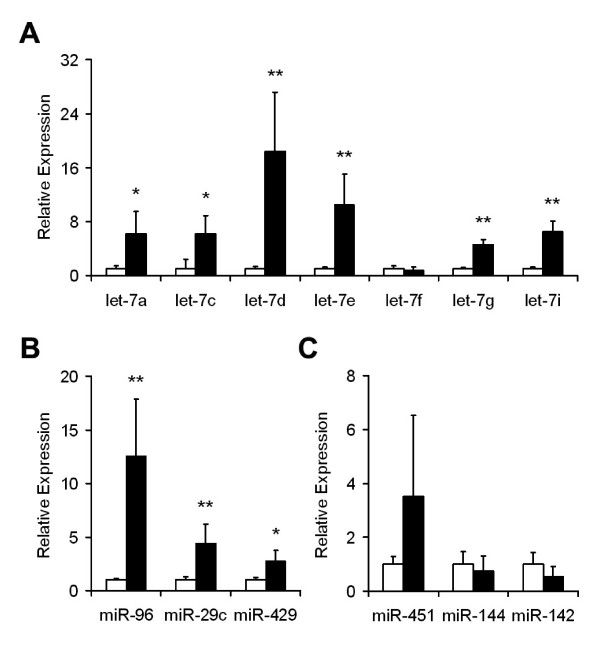
**Validation of miRNA array data using quantitative real-time polymerase chain reaction (qPCR) assay**. **A**. Relative expression patterns for the *let-7 *miRNA that were quantitated by qPCR. Relative expression levels (y-axis) in umbilical cord blood were defined as a level of one for comparison. **B**. Confirmation of miR-96, miR-29c, miR-429 up-regulated expression in adult cells. **C**. Relative expression patterns of the GATA-1 regulated miRNA, miR-451 and miR-144, and hematopoietic tissue-specific microRNA, miR-142. Umbilical cord blood (open bars), adult blood (closed bars), (* *p *< 0.05), (** *p *< 0.01). Note differences in y-axis scales between the three panels.

In order to validate the array-based patterns of human erythroid miRNA, qPCR assays were performed. Relative abundance of miRNA in each sample was calculated by delta Ct method using miR-103 as a reference [14]. Equivalent and high-level expression of miR-103 was detected in cord and adult blood samples (data not shown). The pattern of increased *let-7 *miRNA abundance demonstrated on the arrays was confirmed by qPCR (Figure 2A). Among the *let-7 *miRNA detected on the arrays with significantly increased abundance, *let-7d *and *let-7e *miRNA demonstrated the greatest increases with more than 10 fold increases with qPCR (p < 0.01). Differential expression of *let-7f *was not identified by qPCR, and let-7b failed to amplify. In addition to the *let-7 *miRNA group, qPCR was also used to confirm the expression patterns of other miRNA in these cells. Increased abundance of three other up-regulated miRNA (miR-96, miR-29c, and miR-429) was confirmed (Figure 2B). miR-96 was the most differentially expressed on the arrays, and the qPCR data confirmed greater than a 10-fold increase in adult cells. Up-regulated expression of miR-96 was recently demonstrated in chronic myelogenous leukemia and breast cancer cells [15], and miR-96 may function by regulating expression of the transcription factor FOXO1 [16]. The expression patterns of three other miRNA (miR-451, miR-144, and miR-142) predicted to be expressed in erythroid cells were also examined (Figure 2C). miR-142 is specifically expressed in hematopoietic tissues [17]. The miR-144 and miR-451 genes are known erythroid miRNA that are regulated by the GATA-1 transcription factor [18,19]. All three miRNA species were detected. Adult blood expression of miR-451 was increased, but that increase was not statistically significant.

**Figure 3:**
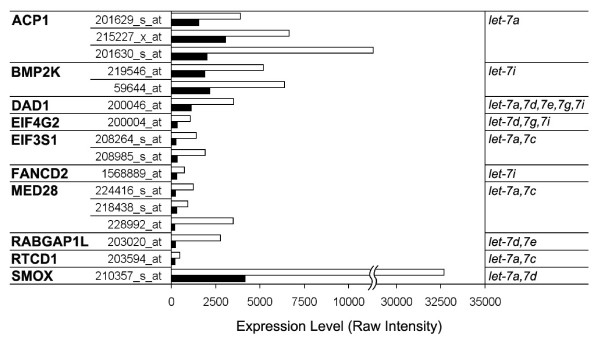
**Reticulocyte mRNA expression levels of 10 genes that are predicted targets of *let-7 *miRNA**. Average intensities of each probe set for *let-7 *target genes in umbilical cord blood versus adult blood were calculated from mRNA expression profiling data using the Affymetrix U133Plus chips. The miRNA predicted to target each gene are shown on the right side of the figure. Umbilical cord blood (open bars), adult blood (closed bars).

While the expression of let-7 genes in human erythroid cells was reported previously [20], this is the first study to demonstrate a developmental increase in the abundance of these gene products. Since *let-7 *miRNA is involved in ontogeny-related gene expression and regulation in lower organisms [8], our study was extended to identify potential mRNA targets of *let-7 *that are expressed in fetal versus adult human erythroid cells. For this purpose, the miRNA expression patterns were combined with mRNA transcriptome analyses. First, miRBase predictions (Version 5) of let-7 major strands were catalogued according to a prediction p-value of less than 0.001. In total, 532 human genes were identified as potential targets of the differentially expressed let-7 miRNA shown in Figure 2. Next, mRNA profiling analyses were performed on the circulating erythroid cells to determine which of the target genes demonstrated down-regulated abundance in the adult cells. Among 532 target genes, the mRNA levels of 10 predicted gene targets were down-regulated in adult blood compared to umbilical cord blood (Figure 3). Collectively, the group includes several genes involved in cellular proliferation (MED28, SMOX) [21,22], and apoptosis (DAD1, EIF4G2) [23,24]. Also, EIF3S1 [25] functions in the 40S ribosomal initiation complex formation, so down-regulation of this non-core subunit of EIF3 may affect erythroblast differentiation or the translational efficiency of globin chain mRNAs [26]. Unlike the model organisms like C. elegans, there was little evidence suggesting let-7 significantly regulates Ras mRNA in these human cells.

This report provides initial evidence that human *let-7 *miRNA, as a group, are up-regulated in association with fetal-to-adult hemoglobin switching. The erythroid focus of this study was chosen due to developmental similarities between fetal-to-adult transition in humans and related developmental changes in lower organisms. Also, miRNA expression patterns during late erythropoiesis were clinically associated with sickle cell anemia and malarial pathogenesis [20,27]. While the results described here may be helpful for generating new hypotheses related to miRNA expression, more robust methods (including coordinated manipulation of multiple miRNA members) are needed to understand the functional significance of increased *let-7 *in adult erythroid cells. We speculate that *let-7 *or other differentially expressed miRNA are involved in the hemoglobin switching phenomenon. Alternatively, the increased *let-7 *expression in adult cells could affect other aspects of erythropoiesis since the predicted target genes are largely involved in cellular proliferation and apoptosis. Overall, these data strongly suggest that miRNA abundance patterns are developmentally regulated in circulating erythroid cells. As such, the data support further erythroid-focused investigation of these curious RNA molecules.

## Conclusion

In addition to globin and other protein-encoding mRNA transcripts [12], miRNA species in circulating erythroid cells are differentially expressed in association with hemoglobin switching. Among the differentially-expressed miRNA, a majority of *let-7 *family members were significantly upregulated in adults. Differential expression of predicted *let-7 *target genes was also detected in the cells. Based upon the importance of *let-7 *for developmental transitions in lower organisms, it is proposed here that differential expression of miRNA including *let-7 *in erythroid cells should be explored for their potential to regulate changes in erythropoiesis or hemoglobin expression patterns in humans.

## Competing interests

The authors declare that they have no competing interests.

## Authors' contributions

SJN and YTL conducted qRT-PCR. SHM and EW carried out miRNA microarray analyses. YTL and SHG performed mRNA profiling in human cord and adult reticulocytes. FMM and DFS assisted in interpreting the data and provided advice on the manuscript. CR collected clinical samples. JLM designed this project. SJN, SHM, and JLM analyzed the data and wrote the manuscript. All authors read and approved the final manuscript.
